# Are Women In Lomé Getting Their Desired Methods Of Contraception? Understanding Provider Bias From Restrictions To Choice

**DOI:** 10.2147/OAJC.S226481

**Published:** 2019-12-05

**Authors:** Elizabeth Pleasants, Tekou B Koffi, Karen Weidert, Sandra I McCoy, Ndola Prata

**Affiliations:** 1 School of Public Health, University of California at Berkeley, Berkeley, CA, USA; 2 Cabinet De Recherche Et D’évaluation (CERA), Lomé, Togo

**Keywords:** provider bias, provider restrictions, contraception, Togo

## Abstract

**Background:**

Despite improvements in contraception availability, women face persistent barriers that compromise reproductive autonomy and informed choice. Provider bias is one way in which access to contraception can be restricted within clinical encounters and has been established as common in sub-Saharan Africa. This analysis assessed the prevalence of provider restrictions and the potential impact on women’s method uptake in Lomé, Togo.

**Methods:**

This sub-analysis used survey data from provider and client interviews collected to assess the impacts of the Agir pour la Planification Familiale (AgirPF) program in Togo. The relationships between provider restrictiveness and women’s receipt of their desired method of contraception were modelled using mixed effects logistic regressions looking at all women and among subgroups hypothesized to be at potentially higher risk of bias.

**Results:**

Around 84% of providers reported a restriction in contraceptive provision for the five contraceptive methods explored (pill, male condom, injectable, IUD, and implant). Around 53% of providers reported restricting at least four of the five methods based on age, parity, partner consent, or marital status. Among all women, there were no significant associations between provider restrictiveness and women’s receipt of desired method, including among those who desired long-acting methods. In adjusted modeling, marital status was a covariate significantly associated with desired method, with married women more likely to receive their desired method than unmarried women (aOR 2.73, 95% CI 1.45–5.13).

**Conclusion:**

Provider reports of high levels of restrictions in this population are concerning and should be further explored, especially its effects on unmarried women. However, restrictions reported by providers in this study did not appear to statistically significantly influence contraceptive method received.

## Introduction

In West Africa, women have both a high and sustained unmet need for modern contraception.^1^ This unmet contraceptive need has been coupled with a surge in population growth in the region.^2^ While family planning policies, including some directly focused on improving access to contraception, have been implemented to address rapid population growth in West Africa,^3^,^4^ the population-level impacts of family planning programs seem to be limited by a variety of challenges related to infrastructure and socio-cultural context, including individual-level barriers.^5^–^7^

Beyond broader challenges in implementing family planning programs, research has also been done to explore the barriers that women face in accessing contraception in West Africa.^1^,^2^ Economic access and/or physical access to clinic (geographic distance); method availability; women’s education and ability to navigate contraceptive decision-making; influences of male partners, specifically partner desire for a large family and/or refusal to use contraception; concern about side effects, misinformation about contraception, and fear; and quality of and satisfaction with care have all been found to be determinants of unmet reproductive care needs in West Africa, playing a role in if and how women could access services.^7^–^9^ In addition, issues related to quality of and satisfaction with sexual and reproductive health care have been explored as a key issue in access with potential for feasible interventions, particularly related to retraining health-care providers to deliver higher quality care. Provider bias in the provision of contraception, or the tendency of health-care providers to deny access to a family planning method as a result of their own prejudices about the method not based in clinical recommendations, is one way in which the quality of care in contraceptive services can be compromised.^10^,^11^

Studies have shown significant provider bias in contraceptive provision in African contexts with providers applying restrictions in the contraceptives they provide based on women’s characteristics. Provider biases potentially contribute to unplanned pregnancy rates and unmet need, particularly for younger, unmarried women who may not receive desired, effective methods of contraception.^11^–^17^ Experiencing bias in contraceptive counseling may result in women not receiving the contraceptive method that they want and may lead to more method discontinuation long-term.^18^–^20^

This analysis will quantify the presence of provider biases in contraceptive provision in Lomé, Togo during the first year of implementing Agir pour la Planification Familiale (AgirPF) program of the US Agency for International Development (USAID)/West Africa and EngenderHealth.^21^ The AgirPF intervention was designed to improve access to and uptake of contraception by addressing supply-side barriers, including quality of clinic services, provider training, and availability of contraceptive resource and services. This study: 1) explores the contraceptive method restrictions reported by providers in the study sample; 2) compares clients who received their desired method of contraception to those who did not; and 3) examines the relationship between provider-reported restrictions in the provision of contraceptive methods and a client receiving her desired method of contraception, with sub-analyses focused on two groups of women thought to be at potentially higher risk of bias—women who desired LARC methods (long-acting reversible contraception, including IUD and implant) and unmarried women.

## Methods

### Data Collection

Data used in this study were collected as part of operations research conducted in Lomé to assess the effectiveness of AgirPF. Data were collected from intervention and control facilities included in the AgirPF baseline data collection. A random sample of 50% was chosen as it would provide sufficient power to assess differences in contraceptive use by clients between intervention and control sites based on the average volume of patients at each clinic per day and the number of days data would be collected at each facility. Sites were selected using the Stata 13 command for random sample selection of half of the universe, without replacement, resulting in 11 intervention and 5 nonintervention facilities in total. These 16 randomly selected sites were located in six different districts in the city and participated in assessments intended to gauge the quality of health-care services provided at the sites. Facility data collection, which included a baseline interview with providers and exit interviews with clients following their clinic visits, was carried out between July and August 2016 in Lomé, Togo.

Provider exit interviews were carried out with 47 providers total, each randomly selected using a lottery system from all midlevel providers working in family planning service provision at that facility (maximum three per facility). The field teams also observed client–provider interactions during family planning consultation and conducted exit interviews for all clients that consented on the days the team was assessing that facility. A total of 1096 facility family planning clients were asked to participate in the study and 1089 clients were interviewed. All clients were women of reproductive age and were generally women with uncomplicated medical histories.

For the purposes of this analysis, data were only included from providers and their clients if the provider reported providing contraception and contraceptive counseling, resulting in 45 eligible providers and 970 client exit interviews. The client exit interviews included further requirements for inclusion: complete demographic information recorded in their exit interview (age, marital status, parity, and education); complete information on the health-care provider; and a reported desired method of contraception (n=619).

### Variable Definition

The primary exposure of interest in this analysis was provider restrictiveness, indicating the provider-reported unnecessary restrictions in the provision of contraception based solely on client characteristics and not medical necessity. Provider restrictiveness was defined as a provider restriction score (range 0–5, 5 being the most restrictive) assigned based on provider self-report of restrictions across contraceptive methods of interest.

In their interview, each provider reported on the restriction of 13 different contraceptive methods by minimum age, maximum age, minimum number of children (parity), partner consent, and marital status. Of the 13 different contraceptive methods in the survey, 5 were of interest in this analysis: combined oral contraceptive pill (the pill), injectable contraception, male condom, intrauterine device (IUD), and implant. For each of these methods, if a provider reported at least one restriction, they were coded as being a restrictive provider for that method (e.g. a provider reported restricting IUDs to only provide them to married women, resulting in a 1 for IUD). The score for restriction of each method was summed to give a provider restriction score, ranging from 0 to 5 for each provider, with a higher score indicating reported restrictions for more methods of contraception (more restrictive); this is a scoring method based on Schwandt et al and modified for the purposes of this analysis.^14^ The provider restriction score was attached to all clients seen by each provider.

The primary outcome of interest was client receipt of desired contraceptive method, among those women with a desired method at baseline. In exit interviews, clients were asked, “Did you come here today to obtain a specific contraceptive method?” “Which method did you want when you came here?” and “Which method did you receive or were you given a prescription or referral for?” Based on these responses, the interviewer indicated “Did the client receive her method of choice?” “Yes” “No” or “Client had no preference at consultation.” For all clients with a “Yes” or “No” response to this question (those who had a desired method before their consultation with the provider), a new dichotomous measure of client receipt of their desired contraceptive method was created. This measure indicated if a client received her method of choice or if she did not. Covariates of interest were determined a priori from the literature on characteristics for which providers have been biased against in similar contexts; these were client age, marital status, parity, and education.

To explore the relationship between the exposure and outcome among women who wanted long-acting reversible contraception (LARC), a client’s desired method of contraception was determined based on the question “Which method did you want when you came?” Additionally, the contraceptive method prescribed or provided to each client was determined based on an additional question in the client exit interview, “Which contraceptive method(s) did you receive or were you given a prescription or referral for?” Each client’s desired method was recoded into a binary variable to explore how the relationships between exposure and outcome might be different looking at women who desired long-acting reversible contraception (LARC) methods (IUD or implant). This resulted in an indicator of if a woman reported an LARC as her chosen method prior to the consultation. To do the same among unmarried women, an indicator of marital status was used that defined married women as those who responded “Married/monogamous” or ‘Married/polygamous” to the question “What is your current marital status?” Women with all other responses (“Living together,” “Single,” “Divorced or separated,” or “Widowed”) were categorized as unmarried.

#### Data Analysis

All analyses were run in StataIC, version 15. Descriptive statistics explored the associations between all covariates and the outcome. To account for the clustering by provider inherent in this data, a mixed-effects logistic regression model was used. This model allowed us to determine the log odds of our outcome of interest (receipt of desired contraceptive method) modeled as a linear combination of provider restrictiveness and any covariates, accounting for the clustering of clients by providers.^22^

We ran separate mixed-effects logistic regressions to examine the relationships between any provider restrictions (dichotomous) and provider restriction score (continuous), with the dichotomous outcome of client receipt of desired method of contraception. We used combined data from intervention and control sites given that results from initial research analyses by study area were not statistically significantly different for the uptake of modern contraceptive methods overall (report to USAID/West Africa, not publicly available). Age, parity, marital status, and education were included in our adjusted models.

There is a focus on improving the uptake of LARC methods in contexts with high fertility rates and low contraceptive use, such as Togo. As a result, we were interested in exploring outcomes for women who came into clinical encounters wanting LARC methods and created a model looking at only those women adjusting for age, parity, marital status, and education (n=132). Additionally, based on what has been found in the past research regarding the importance of client marital status as activator of provider bias, an adjusted model was run restricted to unmarried women (n=92).

### Ethical Approvals

Ethical approval was provided by the Togolese Comité de Bioéthique pour la Recherche en Santé of the Togolese Ministry of Health and Social Protection (Avis N° 017/2016/CBRS du 30 juin 2016). Approval was also provided by the University of California, Berkeley Center for Protection of Human Subjects (CPHS #2016-04-8614). All participants in the study provided informed verbal consent, which was approved by both ethics committees. This sub-analysis of the previously collected data was exempted from review by the University of California, Berkeley Center for Protection of Human Subjects.

## Results

Provider characteristics are summarized in Table 1. All 45 providers were female, while most providers were over 25 years (93.3%), had been at their facility at least a year (71.1%), were midwives (68.9%), and had received at least one in-service training in the past 6 months (71.1%). All providers reported offering injectable contraception, and almost all offered the pill (97.8%) and male condoms (97.8%). A smaller proportion, but still the majority, reported offering LARCs (IUD and implant, 82.2% and 88.9%, respectively). The majority of providers reported at least one restriction in the provision of the five contraceptives of interest (84.4%) and over half reported restricting at least four of the five key contraceptive methods (restriction score = 4 or 5, 33.3% and 20.0%, respectively).

**Table 1:** Descriptive Statistics For All Eligible Medical Providers In Sample Of AgirPF Study Sites (n=45 Providers)

	All Providers
Characteristic	n= 45
Provider sex, n (%)	
Male	0 (–)
Female	45 (100)
Provider age (mean±SD)	36.7 ± 7.6
Provider age (years), n (%)	
Less than 25	3 (6.7)
25–35	18 (40.0)
>35	24 (53.3)
Provider years at facility (mean±SD)	4.9 ±3.9
Provider time at facility (years), n (%)	
Less than 1 year	13 (28.9)
1–3 years	12 (26.7)
4+ years	20 (44.4)
Provider staff type, n (%)	
Midwife	31 (68.9)
Nurse/birth attendant	14 (31.1)
Provider in-service training, n (%)	
No in-service training	19 (42.2)
1–3 in-service trainings	9 (8.9)
4 or more trainings	13 (28.9)
Months since last in-service training, n (%)^a^	
Less than 1 month	19 (42.2)
1–6 months	6 (23.1)
6 or more months	1 (28.9)
Providers offering method of contraception, n (%)	
Combined oral contraceptive pill	44 (97.8)
Injectables	45 (100)
Male condom	44 (97.8)
IUD	37 (82.2)
Implant	40 (88.9)
Providers reporting any restrictions, n (%)	38 (84.4%)
Average total restrictions across all contraceptive methods (possible range: 0–25) (mean±SD)	12.6±9.6
Provider restriction score, n (%)^a^	
0	8 (17.8)
1	5 (11.1)
2	3 (6.7)
3	5 (11.1)
4	15 (33.3)
5	9 (20.0)

**Notes:** ^a^Provider restriction score: This variable is a measure of bias across contraceptive methods, with providers getting an additional point on this score for any report of a restriction for each contraceptive method. Score range: 0–5, for the five contraceptive methods included in this analysis.

Table 2 provides a more detailed view of provider-reported restrictions, showing a breakdown of the type of restriction by a contraceptive method. Age restrictions, where providers reported either a minimum or maximum age for which they would provide a method, were the most commonly reported type of restriction, with the pill most frequently restricted for both minimum and maximum age. Injectable contraception had the highest reports of parity restrictions (33.3% of providers), and IUD and implant both had higher reports of partner consent (both 28.9%) and marital status restrictions (26.7% and 15.6%) than other methods.

**Table 2:** Provider-Reported Contraceptive Restrictions By Type Of Restriction For All Contraceptive Methods Of Interest For All Eligible Medical Providers In Sample Of AgirPF Study Sites (n=45 Providers)

	All Providers
Characteristic	n=45
**Minimum age restriction, n (%)^a^**	**33 (73.3)^b^**
Combined oral contraceptive pill	30 (66.7)
Injectables	28 (62.2)
Male condom	18 (40.0)
IUD	25 (55.6)
Implant	28 (62.2)
**Maximum age restriction, n (%)**	**33 (73.3)**
Combined oral contraceptive pill	32 (71.1)
Injectables	28 (62.2)
Male condom	9 (20.0)
IUD	24 (53.3)
Implant	25 (55.6)
**Any age restriction, n (%)**	**34 (75.6)**
Combined oral contraceptive pill	34 (75.6)
Injectables	34 (75.6)
Male condom	34 (75.6)
IUD	34 (75.6)
Implant	34 (75.6)
**Minimum parity restriction, n (%)**	**23 (51.1)**
Combined oralcContraceptive pill	3 (6.7)
Injectables	15 (33.3)
Male condom	1 (2.2)
Emergency contraception	12 (26.7)
IUD	14 (31.1)
Implant	11 (24.4)
**Partner consent restriction, n (%)**	**21 (46.7)**
Combined oral contraceptive pill	9 (20.0)
Injectables	8 (17.8)
Male condom	2 (4.4)
IUD	13 (28.9)
Implant	13 (28.9)
**Marital status restriction, n (%)**	**19 (42.2)**
Combined oral contraceptive pill	3 (6.7)
Injectables	4 (8.9)
Male condom	1 (2.2)
IUD	12 (26.7)
Implant	7 (15.6)
**Any restriction, n (%)**	**38 (84.4)**
Combined oral contraceptive pill	34 (75.6)
Injectables	32 (71.1)
Male condom	18 (40.0)
IUD	31 (68.9)
Implant	31 (68.9)

**Notes:** ^a^Percent calculated based on n providers that provide the specific method. ^b^Overall value for all contraceptive methods, row percent only included for methods of interest in this analysis and do not average to overall percent as it includes additional providers reporting restrictions for other methods.

Table 3 provides a summary of clients comparing those who received their desired contraceptive method to those who did not, among women who reported a desired method of contraception prior to their consultation (n=619). Married women were much more likely to receive their desired method of contraception compared to unmarried women (χ^2^ =0.001).

**Table 3:** Client Characteristics Associated With Receiving Their Desired Method Of Contraception, Out Of Clients With A Desired Method Prior To Visit In AgirPF Study Sample (n=619 Clients)

Characteristic	All Clients n=619	Clients Did Not Receive Desired Method n=77	Clients Did Receive Desired Method n=542	Test Statistic (p-value)
Client age (mean±SD)	30.0±6.5	28.7±6.3	30.1±6.5	*t*= 1.81 (0.07)
Age (years), n (%)				
<25	123	20 (16.3)	103 (83.7)	*χ*^2^ =0.274
25–35	353	43 (12.2)	310 (87.8)	
>35	143	14 (9.8)	129 (90.2)	
Marital status, n (%)				
Married (monogamous and polygamous)	527	56 (10.6)	471 (89.4)	*χ*^2^ = 0.001
Not currently married	92	21 (22.8)	71 (77.2)	
Parity, n (%)				
Less than 2 children	14	3 (21.4)	11 (78.6)	*χ*^2^ = 0.220
2+ children	129	20 (15.5)	109 (84.5)	
Education level, n (%)				
No education	99	13 (13.1)	86 (86.9)	*χ*^2^ = 0.595
Primary education	201	23 (11.4)	178 (88.6)	
Secondary education	270	32 (11.9)	238 (88.2)	
Higher education	49	9 (18.4)	40 (81.6)	

Results of a mixed-effects logistic regression model showing the relationship between provider restriction score and client receipt of their desired contraceptive method are presented as in Table 4. There was no significant association between provider restriction score and client’s receipt of their desired method of contraception. This model adjusted for education, parity, age, and marital status and the association between provider restriction score and women’s receipt of their desired method remained very close to the null (aOR= 1.09, 95% CI: 0.91–1.32).

**Table 4:** Mixed Effects Logistic Regression Model, Associations Between Receipt Of Desired Method And Provider Restriction Scores With Selected Covariates And Restricted To Women Who Desired LARCs And Unmarried Women In AgirPF Study Sample

	Model 2: Continuous Exposure And Client Receipt Of Desired Contraceptive Method Coefficient (95% CI)		
	Adjusted	Adjusted Among Women Who Desired LARC	Adjusted Among Unmarried Women
Provider restriction score			
0 (ref)	1.00 (ref)	1.00 (ref)	1.00 (ref)
Continuous score	1.09 (0.91, 1.32) p=0.346	1.08 (0.81, 1.44) p=0.620	1.41 (1.02, 1.95)** p=0.036
Education			
No education (ref)	1.00 (ref)	1.00 (ref)	1.00 (ref)
Primary education	1.21 (0.57, 2.59) p=0.622	0.66 (0.16, 2.84) p=0.581	0.75 (0.12, 4.65) p=0.759
Secondary education	1.28 (0.62, 2.68) p=0.500	0.71 (0.17, 2.92) p=0.632	0.37 (0.06, 2.19) p=0.274
Higher education	0.79 (0.28, 2.23) p=0.658	0.75 (0.10, 5.63) p=0.776	0.14 (0.012, 1.71) p=0.125
Parity			
>2 living children	1.00 (ref)	1.00 (ref)	1.00 (ref)
2 or more	1.08 (0.54, 2.16) p=0.821	1.74 (0.43, 7.08) p=0.440	1.52 (0.45, 5.07) p=0.497
Age			
<25 (ref)	1.00 (ref)	1.00 (ref)	1.00 (ref)
25–35	1.19 (0.62, 2.31) p=0.610	0.73 (0.23, 2.31) p=0.596	0.79 (0.22, 2.87) p=0.718
>35	1.52 (0.65, 3.56) p=0.338	0.77 (0.18, 3.32) p=0.732	0.632 (0.10, 3.98) p=0.625
Marital status (all)			–
Unmarried (ref)	1.00 (ref)	1.00 (ref)	
Married	2.73 (1.45, 5.13)*** p=0.002	1.69 (0.52, 5.49) p=0.382

**Notes:** **Significant below 0.05; ***Significant below 0.005.

Results of modeling for the relationship between provider bias score and women’s receipt of their desired contraceptive method adjusted for covariates among women who desired LARC are also presented in Table 4. Looking at only women who reported wanting a LARC method before their consultation (n=132), there was no significant association between exposure and outcome when controlling for education, parity, age, and marital status, and the overall OR stayed essentially the same as the unrestricted adjusted model (aOR= 1.08, 95% CI: 0.81–1.44).

Results of an adjusted model of the relationship between provide restrictiveness score and receipt of desired method among unmarried women are also presented in Table 4. Among unmarried women, when adjusting for education, parity, and age, higher provider bias score was significantly associated with higher odds of receiving their desired method of contraception (p=0.036), with a 41% increase in odds for each unit increase in bias score (aOR= 1.41, 95% CI: 1.02–1.95).

## Discussion

This analysis builds on past research which has explored provider-reported restrictions on contraception, the mix of contraceptive methods used by women, and the experiences of women of different demographic backgrounds in accessing contraception in a variety of contexts in sub-Saharan Africa and beyond. While the previous analyses gave some insight into components of provider bias and contraceptive access, they did not elucidate the link between provider restrictions and women’s access to their chosen methods of contraception. This link is key for understanding how provider bias can be enacted in clinical encounters and how it impacts women’s ability to make contraceptive choices. While the regression results of this analysis suggest no association, this approach to analyzing provider bias is an intuitive progression in this area of research and provides a model for future research. Additionally, the results clarify how measurement can more effectively capture the steps on the pathway between reported restrictions and method outcomes, as well as highlighting some key areas for intervention to improve contraceptive provision in Togo and similar contexts.

Overall, there was a high prevalence of imposed restrictions reported by providers in this sample and many reported restricting multiple contraceptive methods. Age restrictions were the most common type of restriction reported and the IUD and implant were the most restricted methods based on partner-related characteristics, including marital status. Compared to past research done by Sidze et al in urban Senegal, our study sample also reported far more restrictions comparatively.^17^ In their sample of 637 providers at 269 health facilities, the highest proportion of provider reported minimum age restriction was on the pill in public hospitals (59.3%), and for marital status, the implant at public health centers (25.9%). Notably, while the majority of the sample was from urban public facilities similar to those in our study, it also included private facilities which may have contributed to the notable difference in reported restrictions.^17^ In our sample, these maximum values were 66.7% restricting the pill for minimum age and 26.7% restricting the implant for marital status. In an analysis of contraceptive restrictions by 1479 service providers in health facilities in urban Nigeria, Schwandt et al found higher proportions of providers reporting restrictions, with 86.9% restricting the pill for minimum age and 67.3% restricting the IUD for marital status.^14^

While our analysis was in a much smaller sample of providers than previous studies, results seem within the expected range of restrictions reported in this region. From our results, it is clear that many providers reported unnecessary restrictions on contraceptive methods despite having received training intended to promote high-quality counseling. The high prevalence of restrictions across multiple methods highlights a need for additional interventions to address those restrictions and the factors, including technical skills and socio-cultural norms, that could be contributing to them.

Based on modeling, there is no evidence to suggest a significant association between a woman seeing a restrictive provider and receiving her desired method of contraception. The weakness of the relationship between provider restrictiveness and women’s receipt of their desired method of contraception could be due to the fact that the majority of women in this sample did not have characteristics that would make them vulnerable to provider bias; most of the women in this sample were between 25 and 35 years old, married, and had at least two living children. Further data collection efforts and analyses should focus on including young, unmarried, and low parity women and accounting for their experiences in contraceptive counseling, potentially over sampling or targeting this group.

To explore reasons why women were not able to access their chosen methods, we looked at responses to an additional question in the client exit interview “Why do you think you did not get your chosen method?” (full results not presented for this secondary analysis). When looking at the reasons reported, the majority mentioned physical or financial access barriers (too expensive, not available at the clinic, no provider to administer method, n=37) while others commonly reported provider-related reasons (changed mind after listening to provider, preferred method was not appropriate, provider recommended another method, n=24). These reasons indicate that while there was potential provider intervention to discourage clients from using their desired method, appropriate or biased, there were also other supply-side barriers and access issues that kept women from accessing their desired methods. Additionally, the desired method choice could have changed during the consultation due to the woman’s underlying medical conditions contraindicating certain methods.

We found that marital status was an important determinant in the relationship between provider restrictiveness and women’s receipt of their desired method. In our adjusted models looking only at unmarried women, it is unclear why unmarried women with non-restrictive providers were less likely to receive their desired method than those with restrictive providers. But looking at simple proportions, 89.4% of married women received their desired method while only 77.2% of unmarried women did. Restriction and bias by marital status has been found in the past research,^11^,^15^–^17^ supporting our findings of the importance of marital status in this analysis. The influence of marital status on contraceptive provision and the long-term implications of provider biases should be communicated clearly to providers to highlight the need for awareness of bias and the benefits of unbiased counseling.

The strong influence of norms of abstinence before marriage and resulting stigmatization of contraceptive use for unmarried people was already noted by Starling et al.^16^ The importance of marital status in this analysis supports the view that providers place value on women’s marital status when they are accessing contraception, indicating barriers for unmarried women.

The scarcity of young, unmarried, and low parity women in this sample has implications for the generalizability and impact of the outcomes of this analysis. While the majority of this sample was older, married women with children, that is not necessarily representative of the general population of women of reproductive age or of women in need of contraception in Togo. It is very likely that women coming to these clinics were those that wanted modern methods prior, so did not reflect the majority of women in Togo who do not use modern contraception. Additionally, this was a sample of urban and peri-urban clinics in Lomé. Past research has found overall higher proportions of providers in rural areas reporting restrictions compared to urban areas.^23^ Additional exploration of provider restrictions and method outcomes for women in a variety of contexts in Togo is an important component of developing a full picture of provider bias in contraceptive provision.

Measuring women’s receipt of their chosen or desired method of contraception has not been previously explored in similar contexts, and never in conjunction with a measure of provider restrictiveness. A comparable study was carried out in East Java, Indonesia by Pariani et al exploring the effects of contraceptive choice for longer term continuation and found that across all contraceptive methods, 86.3% of women received their chosen method.^24^ We found very similar results, with 87.6% of women receiving their desired method. Our modeling of provider restrictiveness and women’s receipt of their desired contraceptive method builds on the work done by Pariani et al and others in the field to explore different points on the pathway between provider beliefs and client outcomes in the provision of contraception.

While this analysis builds on past research to explore provider bias in a novel way, it has limitations. The exposure indicator used in this study provided opportunities for comparison with the existing body of literature, but they were also subject to social desirability bias that would lend providers to under-report their restrictions, leading to conservative estimates of bias in this sample. It is also not known whether some restrictions are more socially acceptable to report than others, and therefore, more widely reported. The exposure measure used did not capture the method-level biases providers might be enacting, or the nuanced nature of bias as it happens within clinical encounters—a provider reporting restrictions did not mean that they were enacting them in clinical encounters. It is possible that clinical judgement takes over when providers are interacting with women in consultations, lending them to provide methods more widely than they reported they would. It is also possible that women were able to negotiate with providers in clinical encounters, and perhaps those with more social capital, likely married women and/or women of higher parity, were more able to successfully negotiate than those with less power. Further research should be done to explore how reported restrictions differ from those enacted within clinical encounters with women to further elucidate how this occurs and how it might differ by provider and client characteristics.

It should also be noted that the outcome measure used, women’s receipt of their chosen contraceptive method, had limitations. First, womens desired contraceptive method are based on their knowledge of available methods, including their conceptions and misconceptions. Additionally, the use of this outcome indicator means that our regression analyses were restricted to only women who had a desired contraceptive method, leaving women who did not have a desired method out of the analysis. It is plausible that as a result, this analysis may have excluded women with the least reproductive autonomy and contraceptive choice, and therefore, been less sensitive to any enacted biases.

While women’s self-report of receiving their desired contraceptive method had some limitations as our outcome indicator, it did provide the benefit of being closely aligned with client desire in contraception, potentially reducing the influence of provider coercion on the report of the desired method for each woman. Reproductive coercion has been found to occur in a variety of ways in clinical encounters, sometimes by discouraging women from certain methods such as LARCs by emphasizing or inflating adverse effects,^11^ but also by leading women towards LARC methods related to biases or incentivizing their provision. In Sub-Saharan Africa, LARC access is generally still limited by structural factors and programs have largely focused on promoting access and uptake rather than investigating any biases in provision of LARCs.^25^,^26^

Finally, in our sample, it is notable that some women in this study left the clinic with a method other than the one they desired. Additionally, there were women who wanted a method and left without one, and likely women who did not report a desired method but hoped to leave with some form of contraception. While there are structural factors that likely limit access, the potential role of provider bias should be accounted for even if it was not detected in this analysis. Improving contraceptive counseling approaches through interventions that are framed as “provider-aides” rather than programs that emphasize improving the quality of care (implying low existing quality and standards) has been highlighted in past recommendations for addressing provider bias and provides a promising approach to reframing training to engage providers and effectively address biases.^21^ Additionally, carrying out continual supportive supervision with providers within a well-organized system with appropriate supervisors has been advocated an effective approach to ongoing improvement in the quality of contraceptive services.^27^,^28^

## Conclusions

The majority of providers in this study were reporting restrictions in the provision of contraception. Our analysis does not present evidence of any definitive relationship between provider-reported restrictions for women’s ability to access their desired method of contraception for study sites. Even though we found notable methodological limitations that likely compromised our ability to draw any definitive conclusions, it clarifies the needs that exist for further research and improvement in this area. While provider restrictions may not have influenced method outcomes for all women in this study, they are concerning and should be further explored and addressed with a particular focus on the effects of marital status on women’s ability to access their desired methods of contraception. Integrating provider training and appropriate supervision that assesses bias and method outcomes for women into site performance criteria would provide opportunities for quantifying and addressing provider biases. In contexts where improving contraceptive uptake is a priority for governments, eliminating provider bias should also be emphasized.
